# *N*,*N*-Diethyl­anilinium 5-(5-chloro-2,4-dinitro­phen­yl)-2,6-dioxo-1,2,3,6-tetra­hydro­pyrimidin-4-olate

**DOI:** 10.1107/S1600536813004352

**Published:** 2013-02-20

**Authors:** R. Babykala, D. Kalaivani

**Affiliations:** a PG and Research Department of Chemistry, Seethalakshmi Ramaswami College, Tiruchirappalli 620 002, Tamil Nadu, India

## Abstract

In the anion of the title salt, C_10_H_16_N^+^·C_10_H_4_ClN_4_O_7_^−^ [trivial name = *N*,*N*-diethyl­anilinium 5-(3-chloro-4,6,-dinitro­phen­yl)barbiturate], the dihedral angle between the benzene and pyrimidine rings is 45.49 (6)°. The mean plane of the nitro group, which is *ortho*-substituted with respect to the pyrimidine ring, is twisted by 41.57 (13)° from the benzene ring, while the mean plane of the nitro group, which is *para*-substituted, is twisted by 14.41 (12)° from this ring. In the crystal, N—H⋯O hydrogen bonds link cations and anions into chains along [1-10]. Within the chains, inversion-related anionic barbiturate anions form *R*_2_^2^(8) ring motifs.

## Related literature

For different types of inter­actions between electron-deficient nitro aromatics and bases, see: Jackson & Gazzolo (1900 ▶); Mulliken (1952 ▶); Russell & Janzen (1962 ▶); Blake *et al.* (1966 ▶). For donor–acceptor inter­actions see: Mulliken (1952 ▶); Radha *et al.* (1987 ▶). For π–π stacking inter­actions, see: Vembu & Fronczek (2009 ▶). For the biological activity of pyrimidine and barbiturate derivatives, see: Jain *et al.* (2006 ▶); Tripathi (2009 ▶) and of related barbiturates, see: Kalaivani & Buvaneswari (2010 ▶). For the crystal structures of related barbiturates, see: Kalaivani & Malarvizhi (2009 ▶); Buvaneswari & Kalaivani (2011 ▶); Kalaivani & Mangaiyarkarasi (2013 ▶). For hydrogen-bond graph-set motifs, see: Bernstein *et al.* (1995 ▶).
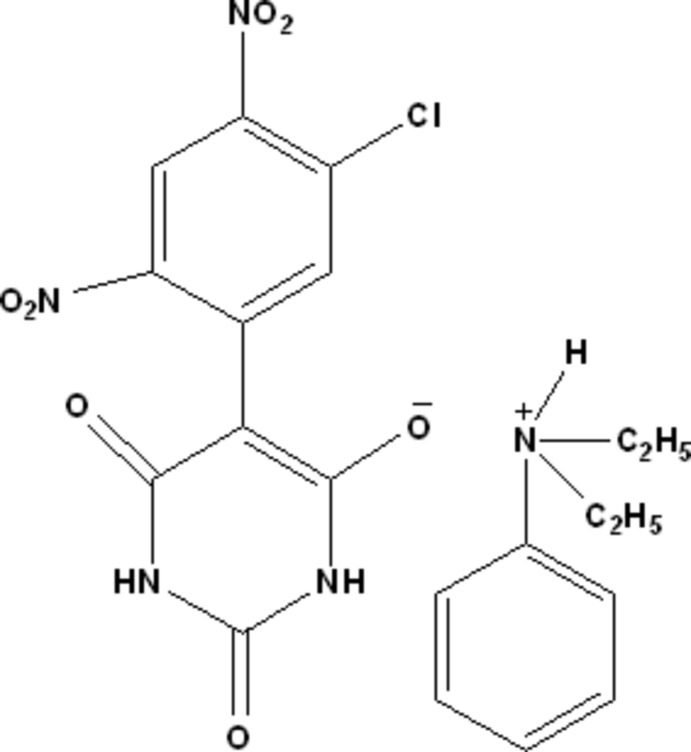


## Experimental

### 

#### Crystal data

C_10_H_16_N^+^·C_10_H_4_ClN_4_O_7_^−^
*M* *_r_* = 477.86
Triclinic, 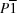

*a* = 9.8040 (2) Å
*b* = 10.2870 (2) Å
*c* = 11.8260 (2) Å
α = 74.727 (1)°
β = 82.761 (1)°
γ = 71.817 (1)°
*V* = 1091.87 (4) Å^3^
*Z* = 2
Mo *K*α radiation
μ = 0.23 mm^−1^
*T* = 293 K
0.30 × 0.30 × 0.20 mm

#### Data collection

Bruker Kappa APEXII CCD diffractometer
Absorption correction: multi-scan (*SADABS*; Bruker, 2004) ▶ *T*_min_ = 0.913, *T*_max_ = 0.985
18678 measured reflections
3836 independent reflections
3123 reflections with *I* > 2σ(*I*)
*R*_int_ = 0.027

#### Refinement

*R*[*F* ^2^ > 2σ(*F* ^2^)] = 0.042
*wR*(*F* ^2^) = 0.117
*S* = 1.04
3836 reflections
312 parameters
H atoms treated by a mixture of independent and constrained refinement
Δρ_max_ = 0.40 e Å^−3^
Δρ_min_ = −0.25 e Å^−3^

### 

Data collection: *APEX2* (Bruker, 2004 ▶); cell refinement: *SAINT* (Bruker, 2004 ▶); data reduction: *SAINT*; program(s) used to solve structure: *SIR92* (Altomare *et al.*, 1993 ▶); program(s) used to refine structure: *SHELXL97* (Sheldrick, 2008 ▶); molecular graphics: *ORTEP-3 for Windows* (Farrugia, 2012 ▶) and *Mercury* (Macrae *et al.*, 2008 ▶); software used to prepare material for publication: *SHELXL97*.

## Supplementary Material

Crystal structure: contains datablock(s) global, I. DOI: 10.1107/S1600536813004352/lh5583sup1.cif

Structure factors: contains datablock(s) I. DOI: 10.1107/S1600536813004352/lh5583Isup2.hkl

Supplementary material file. DOI: 10.1107/S1600536813004352/lh5583Isup3.cml

Additional supplementary materials:  crystallographic information; 3D view; checkCIF report

## Figures and Tables

**Table 1:** Hydrogen-bond geometry (Å, °)

*D*—H⋯*A*	*D*—H	H⋯*A*	*D*⋯*A*	*D*—H⋯*A*
N4—H4*A*⋯O5^i^	0.83 (2)	2.06 (2)	2.892 (2)	175 (2)
N3—H3*A*⋯O7^ii^	0.83 (2)	1.96 (2)	2.794 (2)	180 (3)
N5—H5*A*⋯O6^iii^	0.87 (3)	1.82 (3)	2.677 (2)	168 (3)

Symmetry codes: (i) 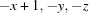; (ii) 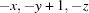; (iii) 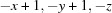.

**Fig. 1.**
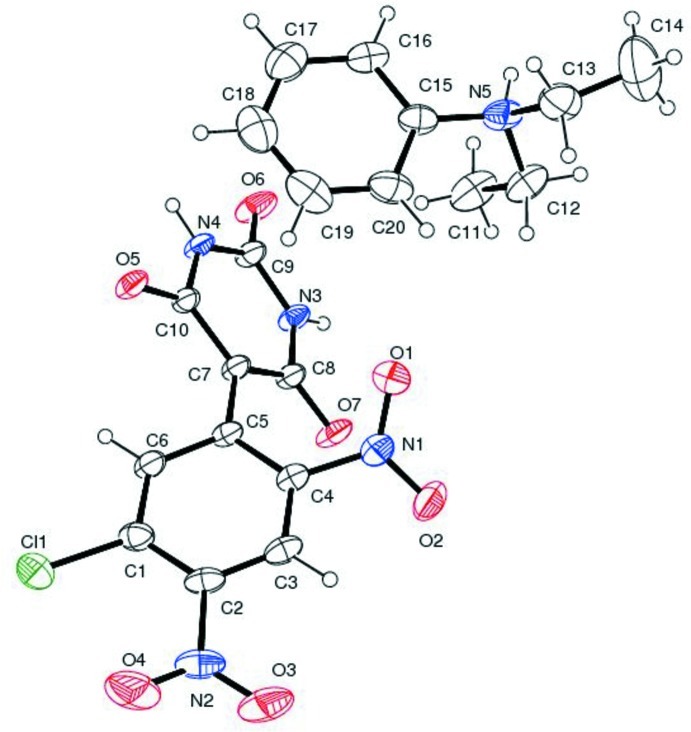
The asymmetric unit of the title compound showing 30% probability displacement ellipsoids.

**Fig. 2.**
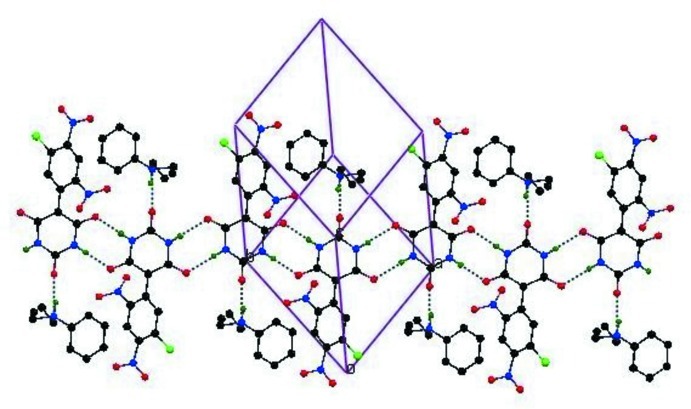
Part of the crystal structure showing the N—H···O hydrogen bonds as dotted lines.

## supplementary crystallographic information

### Comment

Several types of interactions have been observed between electron deficient nitro aromatic compounds and electron rich species (Jackson *et al.*, 1900; Mulliken, 1952; Russell *et al.*, 1962; Blake *et al.*, 1966). Partial transfer of electrons results in the formation of charge-transfer complexes (Mulliken, 1952; Russell *et al.*, 1962). N,N-diethylaniline usually forms charge transfer complexes with electron deficient nitroaromatics which is revealed through the existence of π···π stacking in single crystal X-ray diffraction studies (Vembu *et al.*,2009). The title molecular salt is formed upon mixing 1,3-dichloro-4,6-dinitrobenzene (DCDNB), N,N-diethylaniline and barbituric acid in which no significant π···π stacking is observed between nitro aromatic ring and N,N-diethylaniline ring. Barbituric acid (pyrimidine-2,4,6(1H,3H,5H)-trione) and many other pyrimidine derivatives occupy a distinct and unique place in everyday life (Jain *et al.*, 2006). Barbiturates are mainly used to stop convulsion and they also have hypnotic property which is applied for the treatment of psychotic patients, induction of state of sleep and prolonged sleep (Tripathi *et al.*, 2009). The related barbiturates synthesised in our laboratory also possess such properties (Kalaivani & Malarvizhi 2009; Kalaivani & Buvaneswari 2010). Single crystal X-ray analysis of the molecular salts derived from (1-chloro-2,4-dinitrobenzene/2,4,6-trinitrobenzene), N,N-diethylaniline and barbituric acid have already been reported by our group (Buvaneswari & Kalaivani 2011; Kalaivani & Mangaiyarkarasi, 2013).

The molecular structure of the title compound is shown in Fig 1. In the crystal, N—H···O hyrogen bonds link cations and anions into chains (Fig 2) along [110] which incorporate R^2^_2_(8) rings (Bernstein *et al.*, 1995).

### Experimental

Analytical grade 1,3-dichloro-4,6-dinitrobenzene (DCDNB) and Barbituric acid (BBA) were used as supplied by Aldrich company. N,N-Diethylaniline was distilled under reduced pressure and the fraction boiling over at its boiling point was used. DCDNB (2.37g, 0.01mol) in 15ml of absolute ethanol was mixed with barbituric acid (1.28g, 0.01mol)in 30ml of absolute ethanol. N,N-diethylaniline (3g, 0.01mol) was added to the above mixture, heated to 313K, and shaken well for 5-6 hrs. The solution was kept at room temperature. After a period of two weeks dark reddish orange block-shaped crystals formed in the solution. The crystals were powdered well and washed with 2 to 5ml of ethanol and 50ml of dry ether and recrystallized from absolute alcohol (m.pt :494K ; yield :80 %). Good quality crystals (dark reddish-orange blocks) for single crystal X-ray studies were obtained by slow evaporation of ethanol solution of the title compound at room temperature.

### Refinement

The N-bound H atoms were located in difference Fourier maps and refind independently with isotropic displacement parameters. The C-bound hydrogen atoms were placed in calculated positions and refined as riding atoms: C—H = 0.93, 0.97 and 0.96 Å for CH, CH2 and CH3 H atoms, respectively, with U_iso_(H) = k U_eq_(C), where k = 1.5 for methyl H atoms and = 1.2 for other H atoms.

### Crystal data

**Table:**

C_10_H_16_N^+^·C_10_H_4_ClN_4_O_7_^−^	*Z* = 2
*M**_r_* = 477.86	*F*(000) = 496
Triclinic, *P*1	*D*_x_ = 1.453 Mg m^−^^3^
Hall symbol: -P 1	Mo *K*α radiation, λ = 0.71073 Å
*a* = 9.8040 (2) Å	Cell parameters from 7243 reflections
*b* = 10.2870 (2) Å	θ = 2.2–27.2°
*c* = 11.8260 (2) Å	µ = 0.23 mm^−^^1^
α = 74.727 (1)°	*T* = 293 K
β = 82.761 (1)°	Block, red
γ = 71.817 (1)°	0.30 × 0.30 × 0.20 mm
*V* = 1091.87 (4) Å^3^	

### Data collection

**Table:**

Bruker Kappa APEXII CCD diffractometer	3836 independent reflections
Radiation source: fine-focus sealed tube	3123 reflections with *I* > 2σ(*I*)
Graphite monochromator	*R*_int_ = 0.027
ω and φ scans	θ_max_ = 25.0°, θ_min_ = 2.2°
Absorption correction: multi-scan (*SADABS*; Bruker, 2004)	*h* = −11→11
*T*_min_ = 0.913, *T*_max_ = 0.985	*k* = −12→12
18678 measured reflections	*l* = −14→14

### Refinement

**Table:**

Refinement on *F*^2^	Primary atom site location: structure-invariant direct methods
Least-squares matrix: full	Secondary atom site location: difference Fourier map
*R*[*F*^2^ > 2σ(*F*^2^)] = 0.042	Hydrogen site location: inferred from neighbouring sites
*wR*(*F*^2^) = 0.117	H atoms treated by a mixture of independent and constrained refinement
*S* = 1.04	*w* = 1/[σ^2^(*F*_o_^2^) + (0.0503*P*)^2^ + 0.5116*P*] where *P* = (*F*_o_^2^ + 2*F*_c_^2^)/3
3836 reflections	(Δ/σ)_max_ < 0.001
312 parameters	Δρ_max_ = 0.40 e Å^−^^3^
0 restraints	Δρ_min_ = −0.25 e Å^−^^3^

### Special details

**Table:**

Geometry. All e.s.d.'s (except the e.s.d. in the dihedral angle between two l.s. planes) are estimated using the full covariance matrix. The cell e.s.d.'s are taken into account individually in the estimation of e.s.d.'s in distances, angles and torsion angles; correlations between e.s.d.'s in cell parameters are only used when they are defined by crystal symmetry. An approximate (isotropic) treatment of cell e.s.d.'s is used for estimating e.s.d.'s involving l.s. planes.
Refinement. Refinement of *F*^2^ against ALL reflections. The weighted *R*-factor *wR* and goodness of fit *S* are based on *F*^2^, conventional *R*-factors *R* are based on *F*, with *F* set to zero for negative *F*^2^. The threshold expression of *F*^2^ > σ(*F*^2^) is used only for calculating *R*-factors(gt) *etc*. and is not relevant to the choice of reflections for refinement. *R*-factors based on *F*^2^ are statistically about twice as large as those based on *F*, and *R*- factors based on ALL data will be even larger.

### Fractional atomic coordinates and isotropic or equivalent isotropic displacement parameters (Å^2^)

**Table:**

	*x*	*y*	*z*	*U*_iso_*/*U*_eq_	
C1	0.0071 (2)	0.0230 (2)	−0.27529 (19)	0.0475 (5)	
C2	−0.0626 (2)	0.1317 (2)	−0.36552 (17)	0.0474 (5)	
C3	−0.0487 (2)	0.2634 (2)	−0.38156 (17)	0.0477 (5)	
H3	−0.0941	0.3359	−0.4425	0.057*	
C4	0.0321 (2)	0.2889 (2)	−0.30787 (17)	0.0415 (4)	
C5	0.10006 (18)	0.18534 (19)	−0.21224 (16)	0.0374 (4)	
C6	0.0869 (2)	0.0517 (2)	−0.20168 (18)	0.0436 (5)	
H6	0.1341	−0.0218	−0.1421	0.052*	
C7	0.16905 (19)	0.21325 (18)	−0.12259 (16)	0.0377 (4)	
C8	0.09802 (19)	0.33217 (19)	−0.07694 (17)	0.0402 (4)	
C9	0.2870 (2)	0.2669 (2)	0.05867 (18)	0.0449 (5)	
N4	0.35173 (17)	0.15462 (17)	0.01261 (15)	0.0415 (4)	
C11	0.3494 (3)	0.6406 (4)	−0.1188 (2)	0.0874 (9)	
H11A	0.3532	0.5436	−0.1072	0.131*	
H11B	0.2542	0.6937	−0.0985	0.131*	
H11C	0.4167	0.6476	−0.0699	0.131*	
C12	0.3870 (3)	0.6980 (3)	−0.2442 (2)	0.0762 (8)	
H12A	0.3780	0.7973	−0.2564	0.091*	
H12B	0.3196	0.6896	−0.2933	0.091*	
C13	0.5808 (3)	0.6803 (3)	−0.4067 (2)	0.0747 (7)	
H13A	0.6680	0.6141	−0.4293	0.090*	
H13B	0.5059	0.6876	−0.4564	0.090*	
C14	0.6049 (6)	0.8165 (4)	−0.4284 (4)	0.1406 (17)	
H14A	0.5165	0.8850	−0.4140	0.211*	
H14B	0.6384	0.8432	−0.5085	0.211*	
H14C	0.6756	0.8117	−0.3770	0.211*	
C15	0.5662 (2)	0.4699 (3)	−0.25663 (18)	0.0520 (5)	
C16	0.6740 (2)	0.3853 (3)	−0.1841 (2)	0.0589 (6)	
H16	0.7297	0.4241	−0.1523	0.071*	
C17	0.6985 (3)	0.2419 (3)	−0.1592 (2)	0.0736 (7)	
H17	0.7704	0.1825	−0.1093	0.088*	
C18	0.6163 (4)	0.1865 (3)	−0.2085 (3)	0.0808 (8)	
H18	0.6332	0.0894	−0.1922	0.097*	
C19	0.5097 (4)	0.2736 (4)	−0.2812 (3)	0.0837 (9)	
H19	0.4546	0.2352	−0.3138	0.100*	
C20	0.4836 (3)	0.4154 (3)	−0.3062 (2)	0.0694 (7)	
H20	0.4113	0.4745	−0.3559	0.083*	
N1	0.0461 (2)	0.43164 (19)	−0.33939 (16)	0.0544 (5)	
N2	−0.1569 (2)	0.1174 (3)	−0.44496 (18)	0.0654 (6)	
N3	0.16215 (17)	0.35220 (18)	0.01183 (15)	0.0448 (4)	
C10	0.29895 (19)	0.11832 (18)	−0.07495 (16)	0.0371 (4)	
N5	0.5380 (2)	0.6221 (2)	−0.28078 (16)	0.0585 (5)	
O1	0.16331 (19)	0.44533 (19)	−0.33559 (18)	0.0795 (6)	
O2	−0.05941 (19)	0.52979 (17)	−0.37394 (16)	0.0724 (5)	
O3	−0.2340 (2)	0.2254 (3)	−0.50262 (19)	0.1009 (7)	
O4	−0.1558 (3)	0.0024 (3)	−0.4513 (2)	0.1051 (8)	
O5	0.37052 (14)	0.00914 (13)	−0.10491 (13)	0.0484 (4)	
O6	0.33802 (18)	0.28916 (18)	0.13834 (16)	0.0726 (5)	
O7	−0.01956 (14)	0.41850 (15)	−0.10903 (13)	0.0552 (4)	
Cl1	−0.00919 (8)	−0.14432 (7)	−0.24327 (7)	0.0795 (2)	
H4A	0.429 (3)	0.103 (2)	0.0413 (19)	0.050 (6)*	
H3A	0.120 (2)	0.420 (2)	0.0409 (19)	0.047 (6)*	
H5A	0.590 (3)	0.644 (3)	−0.238 (2)	0.072 (8)*	

### Atomic displacement parameters (Å^2^)

**Table:**

	*U*^11^	*U*^22^	*U*^33^	*U*^12^	*U*^13^	*U*^23^
C1	0.0400 (10)	0.0530 (12)	0.0561 (12)	−0.0093 (9)	−0.0074 (9)	−0.0267 (10)
C2	0.0352 (10)	0.0704 (14)	0.0425 (11)	−0.0100 (9)	−0.0085 (8)	−0.0266 (10)
C3	0.0382 (10)	0.0651 (13)	0.0339 (10)	−0.0044 (9)	−0.0105 (8)	−0.0107 (9)
C4	0.0354 (9)	0.0466 (11)	0.0399 (10)	−0.0052 (8)	−0.0075 (8)	−0.0114 (8)
C5	0.0287 (9)	0.0419 (10)	0.0396 (10)	0.0003 (7)	−0.0082 (7)	−0.0157 (8)
C6	0.0381 (10)	0.0426 (11)	0.0487 (11)	−0.0008 (8)	−0.0153 (8)	−0.0153 (9)
C7	0.0334 (9)	0.0368 (9)	0.0419 (10)	−0.0009 (7)	−0.0133 (8)	−0.0138 (8)
C8	0.0341 (9)	0.0396 (10)	0.0461 (11)	−0.0008 (8)	−0.0134 (8)	−0.0150 (8)
C9	0.0386 (10)	0.0433 (11)	0.0538 (12)	0.0008 (8)	−0.0175 (9)	−0.0212 (9)
N4	0.0323 (8)	0.0398 (9)	0.0508 (10)	0.0047 (7)	−0.0198 (7)	−0.0178 (7)
C11	0.0686 (17)	0.111 (2)	0.0614 (17)	0.0041 (16)	−0.0067 (13)	−0.0204 (16)
C12	0.0681 (16)	0.0864 (19)	0.0613 (16)	0.0003 (14)	−0.0242 (13)	−0.0135 (14)
C13	0.0782 (18)	0.098 (2)	0.0536 (14)	−0.0369 (16)	−0.0194 (13)	−0.0061 (13)
C14	0.221 (5)	0.109 (3)	0.104 (3)	−0.084 (3)	−0.027 (3)	0.008 (2)
C15	0.0459 (11)	0.0741 (15)	0.0426 (11)	−0.0209 (11)	−0.0023 (9)	−0.0205 (10)
C16	0.0425 (11)	0.0852 (17)	0.0492 (13)	−0.0135 (11)	−0.0030 (10)	−0.0218 (12)
C17	0.0590 (15)	0.0819 (19)	0.0656 (16)	−0.0052 (14)	0.0084 (12)	−0.0170 (14)
C18	0.090 (2)	0.0781 (19)	0.0766 (19)	−0.0345 (17)	0.0293 (17)	−0.0260 (16)
C19	0.096 (2)	0.104 (2)	0.0735 (19)	−0.059 (2)	0.0094 (17)	−0.0305 (17)
C20	0.0633 (15)	0.101 (2)	0.0587 (15)	−0.0389 (14)	−0.0094 (12)	−0.0222 (14)
N1	0.0529 (11)	0.0553 (11)	0.0509 (11)	−0.0123 (9)	−0.0147 (9)	−0.0040 (8)
N2	0.0491 (11)	0.1064 (18)	0.0543 (12)	−0.0243 (12)	−0.0102 (9)	−0.0363 (12)
N3	0.0387 (9)	0.0413 (9)	0.0558 (10)	0.0053 (7)	−0.0188 (8)	−0.0267 (8)
C10	0.0336 (9)	0.0348 (9)	0.0431 (10)	−0.0032 (8)	−0.0125 (8)	−0.0127 (8)
N5	0.0554 (11)	0.0807 (14)	0.0446 (10)	−0.0206 (10)	−0.0213 (9)	−0.0142 (10)
O1	0.0631 (11)	0.0739 (12)	0.1013 (14)	−0.0317 (9)	−0.0211 (10)	0.0015 (10)
O2	0.0740 (11)	0.0520 (9)	0.0756 (12)	−0.0005 (8)	−0.0290 (9)	0.0018 (8)
O3	0.0875 (14)	0.1337 (19)	0.0848 (14)	−0.0213 (13)	−0.0548 (12)	−0.0209 (13)
O4	0.1107 (17)	0.1282 (19)	0.1117 (18)	−0.0526 (15)	−0.0386 (14)	−0.0513 (15)
O5	0.0393 (7)	0.0416 (7)	0.0634 (9)	0.0081 (6)	−0.0221 (6)	−0.0258 (7)
O6	0.0622 (10)	0.0751 (11)	0.0873 (12)	0.0129 (8)	−0.0443 (9)	−0.0515 (10)
O7	0.0417 (8)	0.0527 (8)	0.0684 (10)	0.0147 (6)	−0.0284 (7)	−0.0320 (7)
Cl1	0.0822 (5)	0.0605 (4)	0.1107 (6)	−0.0254 (3)	−0.0314 (4)	−0.0274 (4)

### Geometric parameters (Å, º)

**Table:**

C1—C6	1.381 (3)	C12—H12B	0.9700
C1—C2	1.391 (3)	C13—C14	1.444 (4)
C1—Cl1	1.716 (2)	C13—N5	1.511 (3)
C2—C3	1.365 (3)	C13—H13A	0.9700
C2—N2	1.462 (3)	C13—H13B	0.9700
C3—C4	1.371 (3)	C14—H14A	0.9600
C3—H3	0.9300	C14—H14B	0.9600
C4—C5	1.404 (3)	C14—H14C	0.9600
C4—N1	1.463 (3)	C15—C16	1.368 (3)
C5—C6	1.392 (3)	C15—C20	1.376 (3)
C5—C7	1.458 (2)	C15—N5	1.457 (3)
C6—H6	0.9300	C16—C17	1.375 (4)
C7—C8	1.412 (2)	C16—H16	0.9300
C7—C10	1.417 (2)	C17—C18	1.376 (4)
C8—O7	1.247 (2)	C17—H17	0.9300
C8—N3	1.379 (2)	C18—C19	1.367 (4)
C9—O6	1.222 (2)	C18—H18	0.9300
C9—N3	1.348 (2)	C19—C20	1.356 (4)
C9—N4	1.350 (2)	C19—H19	0.9300
N4—C10	1.392 (2)	C20—H20	0.9300
N4—H4A	0.83 (2)	N1—O1	1.208 (2)
C11—C12	1.490 (4)	N1—O2	1.222 (2)
C11—H11A	0.9600	N2—O4	1.201 (3)
C11—H11B	0.9600	N2—O3	1.215 (3)
C11—H11C	0.9600	N3—H3A	0.83 (2)
C12—N5	1.511 (3)	C10—O5	1.239 (2)
C12—H12A	0.9700	N5—H5A	0.87 (3)
			
C6—C1—C2	118.98 (19)	C14—C13—H13B	108.7
C6—C1—Cl1	117.21 (16)	N5—C13—H13B	108.7
C2—C1—Cl1	123.67 (16)	H13A—C13—H13B	107.6
C3—C2—C1	119.82 (17)	C13—C14—H14A	109.5
C3—C2—N2	115.7 (2)	C13—C14—H14B	109.5
C1—C2—N2	124.4 (2)	H14A—C14—H14B	109.5
C2—C3—C4	120.06 (18)	C13—C14—H14C	109.5
C2—C3—H3	120.0	H14A—C14—H14C	109.5
C4—C3—H3	120.0	H14B—C14—H14C	109.5
C3—C4—C5	122.90 (19)	C16—C15—C20	121.9 (2)
C3—C4—N1	114.53 (17)	C16—C15—N5	118.7 (2)
C5—C4—N1	122.53 (17)	C20—C15—N5	119.4 (2)
C6—C5—C4	114.94 (17)	C15—C16—C17	118.7 (2)
C6—C5—C7	120.57 (16)	C15—C16—H16	120.7
C4—C5—C7	124.27 (17)	C17—C16—H16	120.7
C1—C6—C5	123.20 (18)	C16—C17—C18	119.8 (3)
C1—C6—H6	118.4	C16—C17—H17	120.1
C5—C6—H6	118.4	C18—C17—H17	120.1
C8—C7—C10	119.95 (16)	C19—C18—C17	120.2 (3)
C8—C7—C5	118.54 (15)	C19—C18—H18	119.9
C10—C7—C5	121.33 (15)	C17—C18—H18	119.9
O7—C8—N3	117.64 (16)	C20—C19—C18	120.8 (3)
O7—C8—C7	125.00 (16)	C20—C19—H19	119.6
N3—C8—C7	117.34 (15)	C18—C19—H19	119.6
O6—C9—N3	122.27 (17)	C19—C20—C15	118.6 (3)
O6—C9—N4	121.96 (17)	C19—C20—H20	120.7
N3—C9—N4	115.77 (17)	C15—C20—H20	120.7
C9—N4—C10	125.86 (15)	O1—N1—O2	123.6 (2)
C9—N4—H4A	115.2 (15)	O1—N1—C4	118.06 (18)
C10—N4—H4A	118.9 (15)	O2—N1—C4	118.25 (18)
C12—C11—H11A	109.5	O4—N2—O3	122.8 (2)
C12—C11—H11B	109.5	O4—N2—C2	120.0 (2)
H11A—C11—H11B	109.5	O3—N2—C2	117.2 (2)
C12—C11—H11C	109.5	C9—N3—C8	125.13 (16)
H11A—C11—H11C	109.5	C9—N3—H3A	116.8 (15)
H11B—C11—H11C	109.5	C8—N3—H3A	118.1 (15)
C11—C12—N5	112.2 (2)	O5—C10—N4	117.47 (15)
C11—C12—H12A	109.2	O5—C10—C7	126.66 (16)
N5—C12—H12A	109.2	N4—C10—C7	115.85 (15)
C11—C12—H12B	109.2	C15—N5—C13	110.66 (19)
N5—C12—H12B	109.2	C15—N5—C12	113.4 (2)
H12A—C12—H12B	107.9	C13—N5—C12	113.49 (19)
C14—C13—N5	114.3 (3)	C15—N5—H5A	110.8 (17)
C14—C13—H13A	108.7	C13—N5—H5A	105.5 (17)
N5—C13—H13A	108.7	C12—N5—H5A	102.4 (17)
			
C6—C1—C2—C3	1.4 (3)	C17—C18—C19—C20	0.2 (4)
Cl1—C1—C2—C3	177.04 (16)	C18—C19—C20—C15	−0.2 (4)
C6—C1—C2—N2	−176.22 (18)	C16—C15—C20—C19	0.6 (4)
Cl1—C1—C2—N2	−0.6 (3)	N5—C15—C20—C19	−178.9 (2)
C1—C2—C3—C4	−0.9 (3)	C3—C4—N1—O1	−136.1 (2)
N2—C2—C3—C4	176.96 (17)	C5—C4—N1—O1	41.9 (3)
C2—C3—C4—C5	−1.9 (3)	C3—C4—N1—O2	40.2 (3)
C2—C3—C4—N1	176.06 (18)	C5—C4—N1—O2	−141.9 (2)
C3—C4—C5—C6	3.8 (3)	C3—C2—N2—O4	166.9 (2)
N1—C4—C5—C6	−173.95 (17)	C1—C2—N2—O4	−15.4 (3)
C3—C4—C5—C7	−170.75 (18)	C3—C2—N2—O3	−13.0 (3)
N1—C4—C5—C7	11.5 (3)	C1—C2—N2—O3	164.7 (2)
C2—C1—C6—C5	0.8 (3)	O6—C9—N3—C8	−179.2 (2)
Cl1—C1—C6—C5	−175.12 (16)	N4—C9—N3—C8	0.4 (3)
C4—C5—C6—C1	−3.2 (3)	O7—C8—N3—C9	178.2 (2)
C7—C5—C6—C1	171.53 (18)	C7—C8—N3—C9	−0.2 (3)
C6—C5—C7—C8	−130.8 (2)	C9—N4—C10—O5	−178.0 (2)
C4—C5—C7—C8	43.4 (3)	C9—N4—C10—C7	3.3 (3)
C6—C5—C7—C10	44.3 (3)	C8—C7—C10—O5	178.5 (2)
C4—C5—C7—C10	−141.4 (2)	C5—C7—C10—O5	3.4 (3)
C10—C7—C8—O7	−176.7 (2)	C8—C7—C10—N4	−3.0 (3)
C5—C7—C8—O7	−1.5 (3)	C5—C7—C10—N4	−178.07 (17)
C10—C7—C8—N3	1.6 (3)	C16—C15—N5—C13	110.6 (2)
C5—C7—C8—N3	176.84 (18)	C20—C15—N5—C13	−69.9 (3)
O6—C9—N4—C10	177.5 (2)	C16—C15—N5—C12	−120.5 (2)
N3—C9—N4—C10	−2.0 (3)	C20—C15—N5—C12	59.0 (3)
C20—C15—C16—C17	−0.9 (3)	C14—C13—N5—C15	−159.4 (3)
N5—C15—C16—C17	178.5 (2)	C14—C13—N5—C12	71.8 (4)
C15—C16—C17—C18	0.9 (4)	C11—C12—N5—C15	54.0 (3)
C16—C17—C18—C19	−0.5 (4)	C11—C12—N5—C13	−178.6 (2)

### Hydrogen-bond geometry (Å, º)

**Table:**

*D*—H···*A*	*D*—H	H···*A*	*D*···*A*	*D*—H···*A*
N4—H4*A*···O5^i^	0.83 (2)	2.06 (2)	2.892 (2)	175 (2)
N3—H3*A*···O7^ii^	0.83 (2)	1.96 (2)	2.794 (2)	180 (3)
N5—H5*A*···O6^iii^	0.87 (3)	1.82 (3)	2.677 (2)	168 (3)

Symmetry codes: (i) −*x*+1, −*y*, −*z*; (ii) −*x*, −*y*+1, −*z*; (iii) −*x*+1, −*y*+1, −*z*.
